# Overexpression of periostin predicts poor prognosis in non-small cell lung cancer

**DOI:** 10.3892/ol.2013.1590

**Published:** 2013-09-18

**Authors:** LING-ZHI HONG, XIAO-WEI WEI, JIN-FEI CHEN, YI SHI

**Affiliations:** 1 Department of Oncology, Nanjing First Hospital, Nanjing Medical University, Nanjing, Jiangsu 210006, P.R. China; 2 Department of Respiratory and Critical Care Medicine, Jinling Hospital, Nanjing University School of Medicine, Nanjing, Jiangsu 210002, P.R. China

**Keywords:** periostin, non-small cell lung cancer, overexpressed, prognostic factor

## Abstract

The periostin protein, encoded by the POSTN gene, is a component of the extracellular matrix, which is expressed by fibroblasts and has been observed in a variety of human malignancies. The present study aimed to detect the expression of periostin in the tissues of non-small cell lung cancer (NSCLC) patients and benign lung tumors, and to correlate the results with the clinicopathological data of the subjects, in order to evaluate periostin as a potential prognostic marker. In total, 49 NSCLC patients and 6 benign lung tumors were included in this study. The protein level of periostin was detected in paired normal/paratumor/cancer tissues by a western blot analysis and the mRNA level in paired normal/cancer tissues was detected by quantitative polymerase chain reaction (qPCR). The results were then correlated with established biological and prognostic factors. Immunohistochemistry was used to confirm the location of periostin in the NSCLC tissues. Uni- and multivariate analyses were performed using Cox’s proportional hazards regression model. The protein level of periostin was elevated in the cancer tissue of the NSCLC patients compared with the normal (P=0.017) and paratumor (P=0.000) tissues. The expression level in the male patients was much higher than in the female patients at the protein (P=0.001) and mRNA (P=0.010) levels. The mRNA level in the non-adenocarcinoma (non-ADC) patients was much higher than in the adenocarcinoma (ADC) patients (P=0.029). Periostin was demonstrated higher expression at the protein level in the pseudotumors and tuberculosis patients than in the adjacent (P=0.016) and surrounding tissues (P=0.001). Immunostaining indicated that high levels of periostin were present in the mesenchymal areas, but not in the cancer cells themselves. The patients with tumors exhibiting high-level periostin expression showed a significantly shorter survival time (P=0.036, log-rank test). The 3-year survival rate was 81.5% for patients with low-level periostin expression (periostin-L; n=27) and 45.4% for patients with high-level periostin expression (periostin-H; n=22). Similarly, pathological node (pN) status was a significant prognostic marker in the univariate Cox survival analysis. Notably, periostin-H expression was also identified as an independent prognostic factor by the multivariate analysis (P=0.011). These results showed that the overexpression of periostin predicts a poor prognosis, therefore it may be regarded as a novel molecule in the progression and development of NSCLC. The results provide an additional target for the adjuvant treatment of NSCLC.

## Introduction

Lung cancer is the most commonly diagnosed type of cancer (1.6 million cases each year) and the most common cause of cancer mortality (1.18 million mortalities each year). The number of new cases and mortalities is progressively increasing every year. Lung cancer is not easily diagnosed. Only ~10% of lung cancer patients are alive 5 years after diagnosis. Lung cancer is the leading cancer site in males, comprising 17% of total new cancer cases and 23% of total cancer mortalities (1). Non-small cell lung cancer (NSCLC) is the most common type of lung cancer. The development and progression of NSCLC is a complex process, and the tumor microenvironment plays an important role. The tumor microenvironment is composed of structural (extracellular matrix), soluble (cytokines, proteases and hormones) and cellular components (tumor cells, fibroblasts, inflammatory cells, vascular and lymphatic endothelial cells, vascular smooth muscle cells and pericytic cells). In 1993, Takeshita *et al*(2) identified an 811-amino acid protein, named osteoblast specific factor-2 (OSF-2), which was secreted by the mouse MC3T3-E1 osteoblastic cell line. OSF-2 has a typical signal sequence, followed by a cysteine-rich domain (EMI domain), a 4-fold repeated domain and a C-terminal domain. The 4-fold repeated domain shows homology with the insect protein, fasciclin I (2). Each of the 4-fold repeated domains has 150 amino acids, and 90–100 of these 150 amino acids are highly conserved, forming an area called the fas domain. As OSF-2 is also expressed by the periosteum and periodontal ligament, this protein was renamed periostin (3). In humans, the periostin gene (POSTN) is located on chromosome 13, at map position 13q13.3, and the protein is 835 amino acids in size and 90 kDa in molecular weight (4). Mouse and human periostin share 89.2% amino acid identity overall and 90.1% identity in their mature forms.

Periostin is expressed at the mRNA level by the majority of normal adult tissues, including the aorta, stomach, lower gastrointestinal tract, placenta, uterus and breast. The expression of periostin is high in fetal tissue at the mRNA and protein levels (5). Periostin protein expression is observed in normal adult tissues, including the adrenal glands, lung, thyroid, stomach, colon, vagina, ovary, testis and prostate, by western blot analysis (6). Periostin may be induced by transforming growth factor-β (TGF-β) (7) and Bmp-2 (8), and is involved in osteoblast recruitment, attachment and spreading (3). Periostin is considered to be a regulator of cardiac remodeling and hypertrophy and may be a suitable pharmacological target to mitigate heart failure (9).

Recently, periostin was identified as a novel factor in the growth, invasion, angiogenesis and metastasis of numerous types of tumors. Periostin is overexpressed in various types of human cancer tissues, including ovarian cancer (5), cholangiocarcinoma (10), breast cancer (11), colon cancer (12), esophageal cancer (13), head and neck cancer (14), and pancreatic ductal adenocarcinoma (ADC) (15). Notably, periostin expression is well correlated with malignant behavior, including growth, invasion, angiogenesis, metastasis and poor survival in ovarian cancer (5), cholangiocarcinoma (10), breast cancer (11), colon cancer (12), esophageal cancer (14) and pancreatic ductal ADC (15).

Periostin has also been detected in the serum of NSCLC by chemiluminescence assays. Notably, a previous study identified no significant difference between NSCLC patients and normal controls, and there was also no correlation between the serum periostin level and gender, stage, bone metastasis, lymph node status or primary tumor status. However, the NSCLC patients with high periostin levels had significantly poorer survival than the patients with normal periostin levels (16). Periostin mRNA has been shown to be upregulated in NSCLC tissue in relation to normal lung tissue, and also to be correlated with adeno cell subtype and higher tumor grade (17).

However, little information is available on the expression of periostin protein in NSCLC cancer tissues, and the correlation between periostin expression and the clinicopathological characteristics of NSCLC patients is unknown. Previously, we identified that serum periostin was elevated in NSCLC patients compared with normal healthy volunteers, and showed that periostin promotes the proliferation and migration of A549 cells by inducing vimentin and N-cadherin expression and downregulating E-cadherin expression (18). In the present study, the mRNA and protein level of periostin in NSCLC and its correlation with established biological and prognostic factors were investigated. To provide evidence that the inactivation of the periostin gene is a common event in NSCLC, periostin gene expression was examined at the transcriptional and translational levels in 49 paired normal/paratumor/cancer tissues, and the correlation between periostin expression and prognosis in NSCLC was assessed.

## Materials and methods

### Patients

Lung specimens from cancer tissues and paired paratumor tissues (with 1–2 cm distance from tumor edge) and normal tissues (with >5 cm distance from tumor edge) from 49 NSCLC patients (Table I) and 6 benign lung tumors (including 3 inflammatory pseudotumors and 3 pulmonary tuberculosis cases), who underwent pulmonary resection surgery, were included in this study. The samples were obtained by the Department of Cardiothoracic Surgery of Jinling Hospital (Nanjing, Jiangsu, China) between June 2007 and June 2008. All diagnoses were based on pathological evidence. Patients were grouped according to the size of the primary tumor (T), nodal involvement (N) and distant metastasis (M) to TNM stages I–IV according to the World Health Organization criteria for the TNM system and staged appropriately. Patients did not receive chemo-, radio- or immunotherapy prior to surgery. The tissues were snap frozen and stored at −80°C until use. This study was authorized by the principle committee of Jinling Hospital. Written informed consent was obtained from the patients.

### Protein extraction and western blot analysis

Frozen tissues were washed twice with ice-cold phosphate-buffered saline (PBS), and homogenized on ice in 10 volumes (w/v) of lysis buffer [0.1% SDS, 50 mM Tris-HCl (pH 7.5) 1% NP-40, 150 mM NaCl, 1 mM Triton X-100, 1 mM EDTA] containing complete protease inhibitor (PMSF+P8340). Subsequent to incubation on ice for 1 h and centrifugation at 12,000 × g for 15 min at 4°C, the supernatant was collected and stored at −70°C. The protein concentration was measured by the bicinchoninic acid (BCA) protein assay (Sigma, St. Louis, MO, USA). A total of 100 μg protein was separated by 5–10% SDS-PAGE. Proteins were then transferred to polyvinylidene difluoride (PVDF) membranes (Bio-Rad, Hercules, CA, USA), which were saturated by incubation for 2 h with 5% skimmed dry milk in Tris-buffered saline (TBS)/0.05% Tween-20 at 37°C. The membranes were then incubated with the rabbit polyclonal anti-periostin antibody (ab14041, 1:1,000; Abcam, Cambridge, MA, USA) overnight at 4°C in blocking buffer (5% skimmed dry milk in TBS/0.05% Tween-20). Subsequent to being washed 4 times (5 min each) with TBS/0.05% Tween-20, the membranes were incubated with anti-rabbit immunoglobulin labeled with horseradish peroxidase (ab6721, 1:2,000; Abcam) at 37°C in blocking buffer. After 1 h of incubation, the membranes were washed 4 times (5 min each) with TBS/0.1% Tween-20. Chemiluminescence was detected with an ECL western blot analysis detection kit (Pierce Biotechnology, Inc., Rockford, IL, USA), according to the manufacturer’s instructions, and the results were quantified by densitometry using an Image System (GelDoc 2000; Bio-Rad). Polyclonal anti-β-actin (a housekeeping protein used as a loading control to assure equal amounts of protein in all lanes) antibody was used as a control.

### RNA extraction and cDNA synthesis

Total RNA was isolated from the frozen tissue with TRIzol (Invitrogen, Carlsbad, CA, USA). Using random hexamer primers, 2 μg RNA was reverse transcribed to cDNA with a PrimeScript™ 1st Strand cDNA Synthesis kit (Takara Biotechnology, Inc., Shiga, Japan).

### Quantitative polymerase chain reaction (qPCR)

Primers for POSTN and glyceraldehyde-3-phosphate dehydrogenase (GAPDH) were designed and synthesized by Takara Biotechnology, Co., Ltd. (Dalian, China). The basic information of the primers including gene name, National Center for Biotechnology Information (NCBI) reference, forward primer, reverse primer and product size (bp), respectively, were as follows: POSTN, NM_006475, CATTGATGGAGTGCCTGT GGA, CAATGAATTTGGTGACCTTGGTG and 167; and GAPDH, NM_002046, GCACCGTCAAGGCTGAGAAC, TGGTGAAGACGCCAGTGGA and 138. qPCR was performed in triplicate for each sample in a 25-μl reaction mixture, which consisted of template DNA (2 μl), primers (0.2 μM), ROX Reference Dye II (1X), dH_2_O (9.0 μl) and SYBR^®^ Premix Ex Taq [1X; SYBR Premix Ex Taq (perfect real-time) kit; Takara]. PCR was performed using a Stratagene Mx3005P instrument (Stratagene, La Jolla, CA, USA) with the following thermal settings: 1 cycle of 10 sec at 95°C and 45 cycles of 5 sec at 95°C and 20 sec at 60°C. According to the method tested by Tichopad, the relative expression ratio (RR) of the POSTN was calculated based on amplication efficiencies and the cycle threshold comparative with a reference gene (GAPDH) in a sample.

### Immunohistochemistry

Six paraffin sections of tumor were selected for analysis. Immunohistochemical staining was performed on 4-μm formalin-fixed, paraffin-embedded tissue sections. The slides were deparaffinized in xylene and dehydrated in a graded ethanol series. Endogenous peroxidase was blocked with 3% H_2_O_2_ in methanol for 15 min. The primary antibody that was used was the rabbit polyclonal anti-periostin antibody (ab14041, 1:200; Abcam). Immunohistochemical staining was performed using the Envision™ two-step Visualization System (Envision Detection kit GK500705, Peroxidase/DAB, rabbit/mouse; DakoCytomation, Glostrup, Denmark). The next steps were performed according to the manufacturer’s instructions.

### Statistical analysis

The statistical analysis was performed using SPSS 19.0 (SPSS, Inc., Chicago, IL, USA). Values are presented as the mean ± SEM. The significant differences between the tumor, tumor adjacent and surrounding tissues were assessed by the paired samples t-test. The correlation between periostin gene expression and the clinicopathological characteristics of the NSCLC patients was analyzed by the independent samples t-test. The Kaplan-Meier method was used to generate survival curves, and survival differences were analyzed with the log-rank test, based on the status of periostin expression. Uni- and multivariate analyses were performed using Cox’s proportional hazards regression model. P<0.05 was considered to indicate a statistically significant difference.

## Results

### Expression of periostin mRNA and protein in each tissue

**Figure 1:**
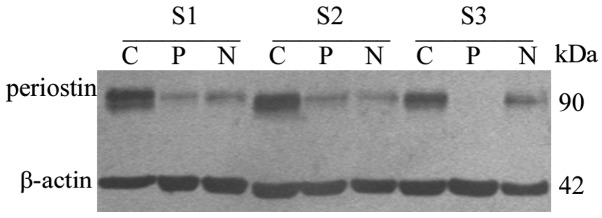
Representative results of periostin protein expression in different lung tissues. Lane C, cancer tissue; lane P, paratumor tissue; lane N, normal tissue. Samples: S1, S2 and S3. kDa, molecular weight.

**Figure 2:**
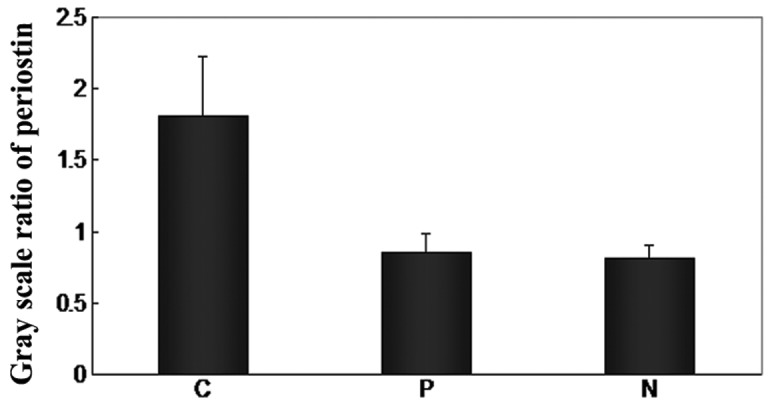
The protein level of periostin in different lung tissues. Lane C, cancer tissue; lane P, paratumor tissue; lane N, normal tissue. The periostin protein gray scale levels of cancer tissues, paratumor tissues and normal tissues were 1.810±0.415, 0.857±0.130 and 0.808±0.100, respectively.

At the mRNA level, the difference between the expression in the cancer tissue and normal tissue was not significant (Table II). The western blot analysis showed that the periostin level was elevated in the cancer tissue of the NSCLC patients (Fig. 1). The periostin protein gray scale levels of cancer, paratumor and normal tissues were 1.810±0.415, 0.857±0.130 and 0.808±0.100, respectively (Table II and Fig. 2). The protein level of periostin in the cancer tissues was significantly higher than in the paratumor (P=0.000) and normal (P=0.017) tissues. However, there were no differences between the paratumor tissues and normal tissues (P=0.978). Periostin expression was also analyzed in 6 benign lung tumors (including 3 inflammatory pseudotumors and 3 pulmonary tuberculosis), and higher expression was observed at the protein level in the pseudotumors and tuberculosis than in the adjacent and surrounding tissues (Table III; P<0.05). In addition, there was no prominent difference between the NSCLC patients and the benign lung tumor patients (data not shown).

### Correlation between periostin expression and the clinicopathological characteristics of NSCLC patients

The correlation between periostin expression and the clinicopathological characteristics of the NSCLC patients was analyzed, and the result is shown in Table IV. As indicated in this table, the expression of periostin had no correlation with age, pathological type, TNM stage, lymph node status, smoking history, tumor size or invasiveness. Periostin gene expression (at the mRNA and protein level) was shown to correlate with the gender of the NSCLC patients; the value of the mRNA and the gray scale level of protein in the male group was 1.438±0.427 and 3.915±0.663, respectively, while those of the female group were 0.449±0.117 and 1.463±0.202, respectively. Statistical significance was determined by the independent samples t-test (Table IV; P<0.05).

### Locating periostin in NSCLC by immunohistochemistry

**Figure 3:**
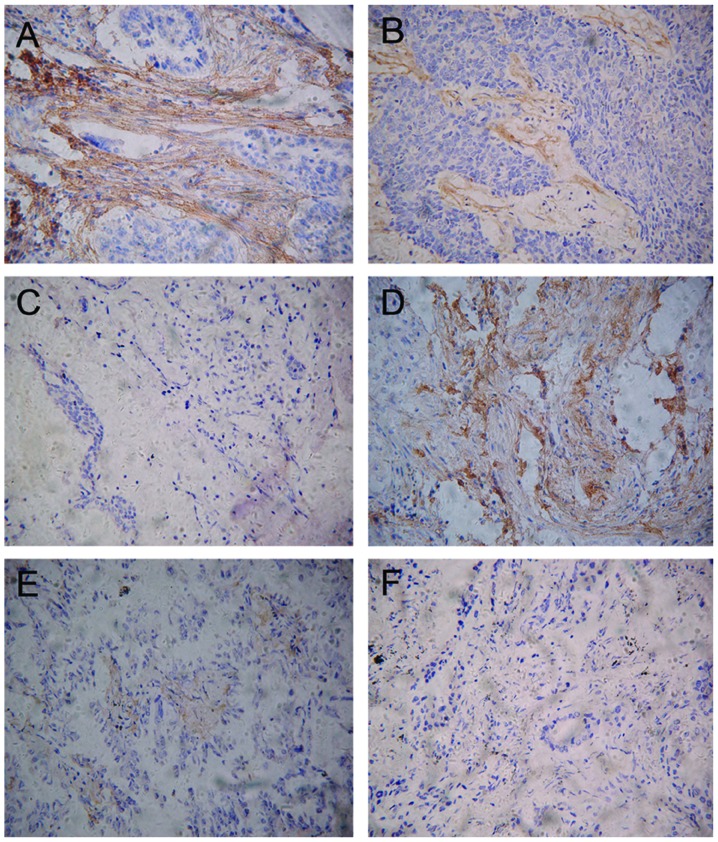
Expression of periostin in non-small cell lung cancer (NSCLC) by immunohistochemistry. (A) Highly-stained squamous carcinoma. (B) Weakly-stained squamous carcinoma. (C) No staining of squamous carcinoma. (D) Highly-stained ADC. (E) Weakly-stained ADC. (F) No staining of ADC. ADC, adenocarcinoma. (Mayer’s hematoxylin stain; magnification, ×200).

To investigate the location of periostin in NSCLC, immunohistochemistry was carried out on 3 ADC slides and 3 squamous carcinoma slides. The immunostaining indicated that high levels of periostin were present in the mesenchymal areas, but not in the cancer cells themselves. Certain samples were highly stained and others demonstrated no staining (Fig. 3).

### Prognostic significance of periostin expression

**Figure 4:**
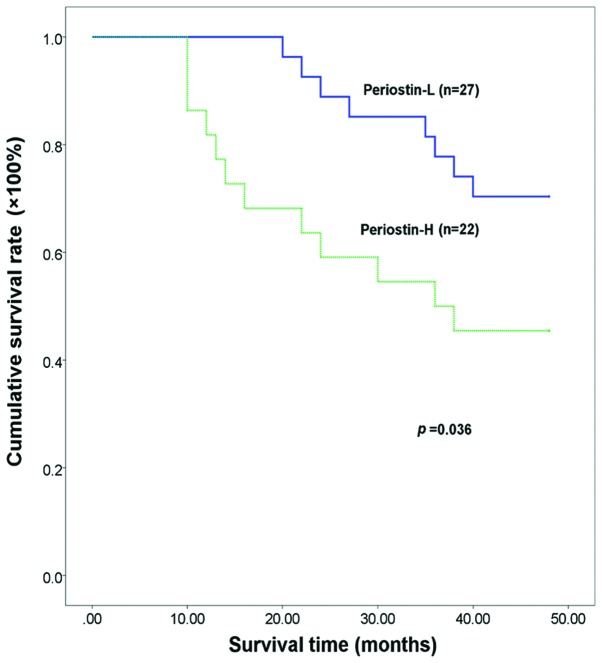
Kaplan-Meier analysis of tumor-specific survival in all non-small cell lung cancer (NSCLC) patients according to periostin expression level. The 3-year survival rate was 81.5% for patients with low-level periostin expression (periostin-L; n=27), and 45.4% for patients with high-level periostin expression (periostin-H; n=22).

A Kaplan-Meier analysis indicated that the NSCLC patients whose tumors showed high levels of periostin expression (periostin-H) had significantly shorter overall survival times compared with those with low levels of periostin expression (periostin-L; P=0.036, log-rank test; Fig. 4). The 3-year survival rate was 81.5% for patients with periostin-L (n=27), and 45.4% for patients with periostin-H (n=22). A univariate analysis was also performed to evaluate the associations between patient prognosis and other factors, including age (<60 vs. ≥60 years), gender (male vs. female), pT status (T1 vs. T2–4), pathological node (pN) status (N0 vs. N1 and N2), histological type (ADC vs. non-ADC), smoking history (smoker vs. never-smoker) and periostin expression (periostin-H vs. periostin-L). Among these parameters, advanced pN status (P=0.044) and periostin status (P=0.044) were significantly associated with a poor prognosis. Since the variables shown to have prognostic affects by univariate analysis may represent covariates, all significant variables from the univariate analysis were included in the multivariate regression analysis in order to identify independent prognostic factors. The resulting data are presented in Table V. Periostin expression was identified as an independent prognostic factor (P=0.011).

## Discussion

Periostin is homologous with fasciclin I, a protein expressed on the surface of a subset of axon pathways in the embryonic central nervous system in insects. Fasciclin I supports cell aggregation and mediates cell sorting, and disruption of fasciclin I causes defects in axonogenesis (19). In mammals, another novel protein that has a similar structure is βig-h3, which was originally cloned as a molecule induced by TGF-β. βig-h3 promotes adhesion and spreading of fibroblasts *in vitro*, and may be associated with microfibrils *in vivo*. Periostin is also a TGF-β-induced extracellular matrix protein involved in cell survival, angiogenesis, invasion and metastasis (20).

Periostin is overexpressed in the tumor tissue of a number of human tumors, and a similar result is shown in the serum of lung cancer (16) and thymoma (21) patients. The mechanism by which periostin interacts with tumors has not been completely elucidated. The majority of analyses have shown that periostin stimulated tumor growth by connecting with integrins, particularly αvβ3, αvβ5 and α6β4. Zhu *et al* demonstrated that recombinant periostin promoted adhesion and migration of epithelial ovarian tumor cells, and that this function was inhibited by the αvβ3 or αvβ5 antibody, indicating that periostin is important in the αvβ3 or αvβ5 integrin-dependent adhesion and migration of epithelial cells (5). Further studies showed that periostin is the ligand of αvβ3 and αvβ5 integrins in breast (11), colon (12) and oral (22) cancer cells. In pancreatic cancer cells, the α6β4-integrin complex acts as the cell receptor of periostin, and this interaction promotes migration through phosphorylation of focal adhesion kinase (FAK) and protein kinase B (AKT) through activation of the PI3 kinase pathway (15). In a previous study, we demonstrated that periostin promotes the proliferation and migration of the human lung ADC cell line (A549) *in vitro* by the EMT pathway (18). Malanchi *et al*(23) showed that periostin was required for cancer stem cell maintenance and that blocking its function prevents metastasis. Periostin recruits Wnt ligands and thereby increases Wnt signaling in cancer stem cells.

In the present study, periostin expression was detected in the tumor, paratumor and normal tissues, and the clinical significance of periostin in the progression and development of NSCLC was observed. The genome was not exactly the same in the paratumor tissue and precancerous lesions, but they were almost identical in histomorphology. When the changes in molecular biology and gene map in the process of tumor progression and development were discussed, the paratumor was selected, and the relative normal tissue from the same patient acted as the control.

Periostin protein levels of the tumor, paratumor and normal tissues of 49 NSCLC patients were detected in the present study. It was demonstrated that the protein level of periostin was much higher in the tumor tissue than in the other 2 groups, but that there was no difference between the paratumor and normal tissues. These findings are similar to the majority of other types of epithelial cancer, including breast (11) and colon (12) cancer. There was no difference between the paratumor and normal tissues at the protein level, therefore periostin was analyzed in the tumor and normal tissue at the mRNA level. However, there was no significant difference between the tumor and normal tissues at the mRNA level. It is well known that mRNA reflects the transcriptional level and protein reflects the translocational level. In the present study, the mRNA level was not consistent with the protein level. The ribosome-loading regulation system and miRNA are likely to play an important role in the translocation of the periostin gene. There are several levels of regulation from transcription to translocation, and mRNAs either degrade or stop translocation in the process. The protein level of periostin is notable with regard to illustrating the function of the gene. Periostin is a novel molecule in the progression and development of NSCLC.

In the present study, the level of periostin was higher in the tumors from the male and non-ADC groups. These findings are also supported by the study of Soltermann *et al*(24), which showed that the high expression of periostin in either the stroma or tumor epithelia was detected in NSCLC tissues by immunohistochemistry, particularly in the male study group. The majority of non-ADC tumors in this study were squamous carcinomas. It is well known that lung squamous cell carcinoma is a male-dominated cancer. Puglisi *et al*(25) showed that periostin was significantly correlated with the expression of the estrogen and progesterone receptors in breast cancer, thus providing a reason why periostin is overexpressed in male and squamous cell carcinoma. However, tobacco smoking may be the most important reason behind this. In the present data, the periostin expression of the patients who had a smoking history (4.350±0.927) was higher than those who did not smoke (2.319±0.446), although there was no statistically significant difference. Tobacco produces >4,000 chemical substances, and 1/80 of them are carcinogenic. Studies have indicated that the polycyclin aromatic and nitroso compounds in the smoke damage the bronchial epithelial cell DNA through a variety of mechanisms, and activate oncogenes (Ras) and deactivate anti-oncogenes (p53). The bronchial epithelial cells transform into cancer cells via this process (26,27). Periostin may be a promoter in this process.

In the present study, periostin was highly expressed in the chronic inflammation patients. It is well known that chronic inflammation may act as tumor promoter. Examples of this may be seen in studies of MALT lymphoma (28) and intestinal carcinogenesis (29). In the present study, there is no direct evidence to reveal the correlation between chronic inflammation and NSCLC. A group of proteins, known as the matricellular proteins, which include thrombospondin (TSP-1 and TSP-2), SPARC (secreted protein acidic and rich in cysteine), osteopontin (OPN), the tenascins (TN-C and TN-X) and the CCN family members (CCN1–6), are known to be expressed at lower levels in normal adult tissue, but are upregulated during wound healing and tissue remodeling. The expression of these proteins is also involved in tumor development and progression. In normal situations, these proteins are well controlled by various signals. However, in tumors, the expression of the matricellular proteins is defined in a deregulated manner as a ‘wound that does not heal’ (30). In the present study, periostin was upregulated in the NSCLC and chronic inflammation patients, therefore we hypothesize that periostin may be a member of the matricellular protein family and that it has an effect on chronic inflammation and cancer. Periostin may be a suitable target to block the dangerous loop between cancer and inflammation.

Using immunohistochemistry, the present study showed that periostin was only located in the mesenchymal tissue surrounding the tumor cells. This corresponded to the location of periostin previously identified in other types of cancer cells (5,6,11,12). In the present study, the pattern of the localization of periostin in the tumoral stroma appears typically fibrillar and branched, suggesting a possible association between periostin and the fibers of the desmoplastic stroma of NSCLC. Recently, immunoelectron microscopy analyses of mouse periodontal ligaments has revealed a close association between periostin and collagen fibers, indicating that a similar association may take place in NSCLC tissues (31).

The localization and function of periostin in the juxtatumoral stroma exhibits similarities to other secreted proteins, such as the CCN family of proteins. The CCN family is a group of 6 secreted proteins that are specifically associated with the extracellular matrix. Similar to periostin, CNN family members are induced by growth factors and cytokines, such as TGF-β and endothelin 1, and cellular stress, including hypoxia, and are overexpressed in pathological conditions that affect connective tissues, including scarring, fibrosis and cancer. They also interact with integrins and act as matricellular proteins to mediate cell adhesion, migration, tissue repair (32), fibrosis (33), cancer and vascular disease (34).

For the present survival analysis, the 3-year overall survival rate for the patients with periostin-L expression was much higher than for those with periostin-H expression. Furthermore, the multivariate analysis revealed that periostin-H expression was an independent prognostic factor. The results indicate that periostin is a crucial prognostic factor. These findings lead us to believe that the overexpression of periostin is likely to represent an important transformation factor associated with a more malignant phenotype in NSCLC patients.

In conclusion, although the present study involved only 49 patients, it may be concluded that periostin is important in the progression and development of NSCLC. There was an abnormally high expression level of periostin in the NSCLC and lung chronic inflammation patients, and the periostin expression level was much higher in the male and non-ADC groups of NSCLC patients. We considered that periostin may be pivotal in the pathogenesis and development of NSCLC, and that chronic inflammation may promote cancer development using certain molecules, including periostin. It was demonstrated that periostin was only located in the juxtatumoral stroma of the NSCLC tissues, along with those proteins belonging to the CCN family members, which had been shown to be integrin-dependent. Patients with high level of periostin achieved a significantly inferior outcome, indicating that it is a malignant phenotype in NSCLC.

## Figures and Tables

**Table I:** Characteristics of the patients with primary non-small cell lung cancer (western blot analysis and qPCR).

Characteristics	Value
Total patients, n (%)	49 (100)
Age, years^a^	57.132±1.743
Gender, n (%)	
Male	38 (77)
Female	11 (23)
Age, n (%)	
<60 years	27 (55)
≥60 years	22 (45)
Histological type, n (%)	
ADC	35 (71)
Non-ADC	14 (29)
Stage, n (%)	
I	15
II	10
I+II	25 (51)
III	23
IV	1
III+IV	24 (49)
Tumor size and invasiveness, n (%)	
T1	11
T2	28
T1+T2	39 (79)
T3	5
T4	5
T3+T4	10 (21)
Lymph node status, n (%)	
+	25 (51)
−	24 (49)
Smoking history, n (%)	
≥20 pack year	25 (51)
<20 pack year	24 (49)

a Data are presented as the mean ± SEM.

qPCR, quantitative polymerase chain reaction; ADC, adenocarcinoma.

**Table II:** Expression of periostin mRNA and protein in three types of tissues of NSCLC patients.

Group	Periostin mRNA	P-value	Periostin protein	P-value
Cancer tissue	0.326±0.086	-	1.810±0.415	-
Paratumor tissue^a^	-	-	0.857±0.130	0.000^b^
Normal tissue	0.389±0.085	0.433^c^	0.808±0.100	0.017^c^

a At the mRNA level, periostin expression was not detected in the paratumor tissue. Data, with the exception of P-value, are presented as the mean ± SEM.

Periostin expression:

b Difference between cancer tissue and paratumor tissue;

c difference between cancer tissue and normal tissue. NSCLC, non-small cell lung cancer.

**Table III:** Expression of periostin mRNA and protein in three types of tissues of benign lung tumors.

Group	Periostin mRNA	P-value	Periostin protein	P-value
Tumor tissue	0.237±0.077	-	1.178±0.160	-
Adjacent tissue^a^	-	-	0.520±0.161	0.016^b^
Surrounding tissue	0.460±0.230	0.378^c^	0.235±0.111	0.001^c^

a At the mRNA level, periostin expression was not detected in the adjacent tissue. Data, with the exception of P-value, are presented as the mean ± SEM.

Periostin expression:

b Difference between tumor tissue and adjacent tissue;

c difference between tumor tissue and surrounding tissue.

**Table IV:** Expression of periostin in NSCLC cancer tissue and its correlation with clinicopathological characteristics of NSCLC patients.

Parameter	Periostin mRNA	P-value	Periostin protein	P-value
Gender				
Male	1.438±0.427	0.010	3.915±0.663	0.001
Female	0.449±0.117		1.463±0.202	
Age, years				
<60	0.742±0.104	0.085	2.468±0.490	0.075
≥60	1.913±0.809		4.663±1.060	
Pathological type				
ADC	0.669±0.096	0.029	3.068±0.665	0.566
Non-ADC	1.939±0.754		3.701±0.897	
TNM stage				
I+II	0.982±0.193	0.534	3.688±0.880	0.543
III+IV	1.403±0.612		3.016±0.649	
Lymph node status				
+	1.347±0.557	0.621	3.859±0.759	0.255
−	1.008±0.216		2.613±0.708	
Smoking history				
≥20 pack year	1.159±0.197	0.895	4.350±0.927	0.059
<20 pack year	1.249±0.645		2.319±0.446	
Tumor size and invasiveness				
T1+T2	0.997±0.148	0.538	3.356±0.684	0.948
T3+T4	1.784±1.222		3.275±0.723	

Data, with the exception of P-value, are presented as the mean ± SEM. NSCLC, non-small cell lung cancer; ADC, adenocarcinoma.

**Table V:** Prognostic factors in Cox’s proportional hazards model.

Variables	Risk ratio	Univariate 95% CI	P-value	Risk ratio	Multivariate 95% CI	P-value
Age, years						
<60/≥60	1.635	0.680–3.933	0.272	2.425	0.857–6.859	0.095
Gender						
Male/female	0.541	0.159–1.849	0.328	0.962	0.207–4.460	0.960
pT status						
pT2–4/pT1	0.329	0.076–1.420	0.136	0.416	0.084–2.061	0.282
pN status						
pN1–2/pN0	0.353	0.128–0.974	0.044^a^	0.431	0.152–1.224	0.114
Histological type						
Non-ADC/ADC	0.448	0.185–1.084	0.075	0.448	0.153–1.316	0.144
Smoking history						
Smoker/non-smoker	0.708	0.289–1.733	0.450	0.987	0.335–2.905	0.981
Periostin expression						
Periostin-H/-L	0.399	0.163–0.977	0.036^a^	0.251	0.087–0.724	0.011^a^

a Significance: CI, confidence interval; ADC, adenocarcinoma; POSTN, periostin; pT, pathological primary tumor; pN, pathological node.
